# Transition from child to adult health services for young people with cerebral palsy in Ireland; implications from a mixed-methods study

**DOI:** 10.12688/hrbopenres.13912.1

**Published:** 2024-07-17

**Authors:** Jennifer M. Ryan, Meriel Norris, Aisling Walsh, Amanda Breen, Owen Hensey, Claire Kerr, Sebastian Koppe, Grace Lavelle, Mary Owens, Michael Walsh, Thilo Kroll, Jennifer Fortune

**Affiliations:** 1 CP-Life Research Centre, School of Physiotherapy, RCSI University of Medicine and Health Sciences, Dublin, Ireland; 2 College of Health, Medicine and Life Sciences, Brunel University London, London, England, UK; 3 School of Population Health, RCSI University of Medicine and Health Sciences, Dublin, Ireland; 4 Central Remedial Clinic, Dublin, Ireland; 5 School of Nursing and Midwifery, Queen's University Belfast, Belfast, Northern Ireland, UK; 6 Enable Ireland, Galway, Ireland; 7 Institute of Psychiatry, Psychology & Neuroscience, King's College London, London, England, UK; 8 UCD IRIS Centre, School of Nursing, Midwifery and Health Systems, University College Dublin, Dublin, Leinster, Ireland

**Keywords:** cerebral palsy, disability, transition, health services, mixed-methods

## Abstract

**Background:**

Poor transition from child- to adult-oriented healthcare may lead to negative outcomes and dissatisfaction with services in adulthood. The aim of the study was to examine how transition is provided to and experienced by young people with CP in Ireland. This report provides integrated quantitative and qualitative findings and implications based on the totality of knowledge generated.

**Methods:**

A convergent parallel mixed-methods study was conducted. Data were collected from people with CP aged 16-22 years, parents, and health professionals using surveys and semi-structured interviews, informed by a framework of nine key transition practices. Quantitative and qualitative findings were integrated at the interpretation stage of the research using integration through joint displays. Implications were developed through discussions with health professionals, young people, and parents.

**Results:**

Surveys were completed by 75 young people/parents and 108 health professionals. Interviews were conducted with 13 young people, 14 parents, and 27 health professionals. There was complementarity between quantitative and qualitative findings indicating lack of a named worker, limited information provision, insufficient self-management support, no opportunity to meet the adult team, limited contact with the GP, and no opportunity for attending formal life skills training. There was dissonance between quantitative and qualitative findings regarding appropriate level of parental involvement. There was silence between quantitative and qualitative findings for promotion of health self-efficacy and senior manager responsible for transition, with quantitative findings indicating these were not present for most young people, but qualitative findings not describing these practices

**Conclusion:**

Implications of integrated findings include the need for a standardised transition pathway, intentional actions to enable parents and young people to adapt to changing roles, provision of information in a collaborative and phased approach, a common understanding of self-management between young people, parents and health professionals, and the need to involve adults’ services and GPs in transition.

## Introduction

In this paper, we report the integrated findings from the Ignition study, a mixed-methods study to examine how transition care is provided to and experienced by young people with cerebral palsy (CP) in Ireland ^1^. In-depth findings from quantitative and qualitative studies are separately reported elsewhere ^2, 3^. The purpose of this paper is to enhance the value of the study by integrating quantitative and qualitative findings and to provide implications based on the totality of knowledge generated.

Cerebral palsy (CP) is one of the most prevalent disabling conditions among children worldwide. Children with CP require transfer from child-centred to adult-oriented healthcare, typically between the age of 16 and 18 years. Transfer is the event when responsibility for healthcare shifts from a child to adult healthcare provider. After transfer to adult-oriented healthcare, many adults with CP experience difficulty navigating services ^4^, reduced access and visits to specialist services, and a decrease in use of rehabilitation services ^4, 5^. Poor preparation for this transfer may contribute to the deterioration in function and development of chronic conditions ^6, 7^.

Transition is defined as ‘the purposeful, planned process that addresses the medical, psychosocial, educational and vocational needs of adolescents and young adults with chronic medical and physical conditions as they move from child-centred to adult-oriented healthcare systems’ ^8^. Although guidance to support transition has been published in several countries ^9– 11^, there is still evidence that young people with CP and parents are not prepared for transition, lack support and information, and struggle to navigate the adult healthcare system ^5^.

To facilitate the implementation of successful transition for young people with CP, it is important to understand the experience of transition from multiple perspectives of young people with CP, parents and health professionals. The Ignition study firstly assessed gaps in the current management of transition against best practice from the perspective of service users (young people and parents) and service providers. Secondly, it explored transition experiences from the perspective of young people with CP, their families and health professionals.

The aim of this study is to explore the experience of transition and implications for practice by integrating quantitative and qualitative findings.

## Methods

We conducted a convergent parallel mixed methods study ^1^ and collected quantitative and qualitative data from young people with CP aged 16–22, parents, and health professionals. We used convenience and snowball sampling. We shared information about the study through three national organisations that provide health and social care services to people with CP, disability officers in higher education, special education needs schools, professional bodies, and social media. Data collection and analysis are described in detail elsewhere ^2, 3, 12^. Data collection and analysis were informed by a framework of nine key transition practices representing best practices, which is published in the study protocol ^1^ and presented in Box 1. These transition practices were based on a research programme conducted in the UK ^13^. the UK NICE guideline on transition ^9^ and national strategies relating to children and neurodisability in Ireland ^14– 17^.

**Box 1. Description of nine key transition practices**

**Table:** 

**Named worker:** A named worker, known to the young person, oversees, co-ordinates or delivers transition support and acts as the link between the young person and the various practitioners involved in their care, including their GP. This person may not be formally allocated and may not be a health provider but should remain in close contact with health services.
**Appropriate parent involvement:** Involvement of parent(s)/carer(s) in the young person’s care at a level that is deemed appropriate by both the young person and parent(s)/carer(s)
**Information:** Child and adult services provide young people and families with information that describes the transition process and the support available before and after transfer. The information should specifically mention health services, which encompasses services that directly impact people's physical health, mental health, and social well-being. This information should be provided early enough to allow young people time to reflect and discuss with parent(s)/carer(s) or health professionals and be in an accessible format. Where there is no adult service for a young person to transfer to, information about known and trusted voluntary organisations who could provide support should be provided to the young person.
**Promotion of health self-efficacy:** Promotion of health self-efficacy (ie, actively helping young people to feel confident in managing their condition), where health encompasses complete physical, mental and social wellbeing, including provision of information to the young person about their condition and encouragement to take responsibility for their health.
**Self-management support:** Promotion of opportunities for self-management, where the individual is directly involved in planning and decision-making around their needs and takes responsibility for maintaining optimal physical, mental and social wellbeing.
**Meet the adult team:** A health professional from the relevant adult services or primary care meets the young person before they transfer from child services
**Senior manager:** A senior manager with responsibility for implementing transition strategies and policies, including facilitating good working relationships between child and adult services, ensuring appropriate materials are available, and monitoring that the person has a suitable appointment in adult services. This person may not be known to the young person
**Discharge letter to GP:** Where there is no adult service for a young person to transfer to, a detailed discharge letter is sent to the young person’s GP.
**Formal life-skills training:** Formal training, relevant to health condition, in wider life skills—education, gaining employment, finances, housing, social relationships, sexual health, mental health. The health service may not provide such training, but during consultations, staff should inquire about such matters and make referrals to other agencies as needed.

Quantitative data were collected using two questionnaires completed by 75 young people and/or parents and 108 health professionals ^2^. We collected data on socio-demographic and condition-specific data, and the experience or provision of transition practices. Descriptive statistics (e.g. mean, SD) were used to describe data as appropriate. Qualitative data were collected using in-depth semi-structured interviews with 13 young people, 14 parents, and 27 health professionals ^3, 12^ and analysed using the Framework Method. Indicative questions are included in Table S1. 

This study obtained approval from the research ethics committee of the Principal Investigator’s host institution and the research ethics committees of two organisations that supported recruitment (Royal College of Surgeons in Ireland's Research Ethics Committee, REC201911010, 6 ^th^ February 2020; Enable Ireland Research, Ethics and Quality Committee, RA72JR2020, 16 ^th^ September 2020; Central Remedial Clinic Research Ethics Committee, 73001, 9 ^th^ July 2020. Written informed consent was not obtained from those who completed the online questionnaire because it was anonymous and completion indicated consent. However, before completing the anonymous online questionnaire, participants confirmed they had read and understood the information leaflet, knew they could withdraw at any time, and were happy to complete the questionnaire. Where a person completed a paper questionnaire, they provided written informed consent. Where the person was under the age of 18 years, their parent or guardian additionally provided written consent for their child to participate. Written or verbal informed consent was obtained from young people, parents, and health professional to participate in interviews, as approved by the ethics committees.

### Integration

We integrated quantitative and qualitative data at the interpretation stage of the research using integration through joint displays ^18^. We produced a convergence coding matrix, which displayed quantitative and qualitative findings relating to each key transition practice in the framework (Table S2). Qualitative findings are presented according to themes from two individual papers ^3, 12^. Co-authors (JR, JF, MN, AW) discussed the matrix and considered where there was agreement and disagreement between findings, defined as convergence (i.e., findings agree directly), complementarity (i.e., findings offer complimentary information), dissonance (i.e., findings seemingly contradict each other) or silence (i.e., themes arise in quantitative data or qualitative data but not both). Additionally, we identified the key implications of our integrated findings through discussions with the wider research team and our advisory groups of health professionals, young people, and parents. Therefore, in this paper, we report the integrated findings and present implications of this research.

## Results

We outline integrated findings for each transition practice according to whether there was complementarity, dissonance or silence between quantitative and qualitative findings.

### Complementarity between quantitative and qualitative findings

There was complementarity between quantitative and qualitative findings for the following practices.

***Named worker***

Quantitative and qualitative findings indicated lack of a named worker to oversee, co-ordinate or deliver transition support. Most young people did not have a named worker, and most health professionals stated their service did not ensure each young person had a named worker for transition. Limited time and high staff turnover restricted health professionals’ ability to support young people with transition within their existing roles. Within some services, an individual with an interest and passion for transition took on the role of a named worker, resulting in variation within and between services. This became unsustainable when the person did not have dedicated time to fulfil the role. In the absence of a dedicated named worker for transition assigned to young people, staff also lacked clarity about who was responsible for supporting transition.

***Information provision***

Quantitative and qualitative findings indicated young people and families received limited information describing the transition process and the support available before and after transfer. Young people who had been discharged from children’s services were more likely to receive information than those still attending children’s services. Receiving information immediately before discharge, or in many cases not at all, prevented young people from engaging in transition and resulted in young people and parents feeling abandoned. Health professionals often provided information when asked and when they perceived it was needed. They saw their role as facilitative rather than prescriptive. Further, health professionals sometimes withheld information to protect the young person and family from a lack of equivalent health services in adulthood. They also hesitated to discuss the young person’s condition or medical history with them if they thought parents had not yet shared this information. In contrast, young people often preferred to receive information directly and found it difficult to find trusted sources of information. Young people and parents also had specific information needs and knowledge gaps that covered a breadth of topics, including the transition process, their CP, service provision in adulthood, managing their CP in adulthood, and education and living options, which may be beyond one professional’s expertise.

***Self-management support***

Quantitative and qualitative findings indicated that young people did not receive enough support to self-manage their physical and mental health. Although most health professionals stated they or their service supported young people to take responsibility for maintaining optimal health, very few had a written protocol for this. Qualitative findings suggest that young people and health professionals have different perspectives on self-management. Young people received support to manage specific aspects of their care, such as completing an exercise programme. However, they wanted support to develop a range of skills and knowledge to enable them to manage their condition as adults, such as talking to health professionals, making appointments, and identifying when and how to access health services. They often lacked time, space and opportunities in healthcare settings to practise and develop skills. Their lack of knowledge about their condition, medical history and potential changes in their condition as they age also negatively impacted their ability to self-manage. Health professionals provided space for young people to develop self-management skills. However they often did not explicitly make the young person aware of this, which resulted in the young person feeling unsupported.

***Meet the adult team***

Quantitative and qualitative findings indicated young people did not have the opportunity to meet with someone who provided their healthcare in adulthood before transfer. Qualitative findings indicated a lack of equivalent multidisciplinary services for adults with CP, which resulted in health professionals in children’s service not knowing if the young person would receive a service in adulthood or where to refer them. If they identified a service for the young person, they often did not know which professional within the service would see the young person. Many adults with CP accessed community-based or primary care services in adulthood, which typically accepted people with an acute need only. Therefore, it was impossible to directly transfer the young person from children’s services. These services also typically lacked expertise about the needs of adults with CP and were sometimes unwilling to engage with adults with CP. The lack of adult services and uncertainty about referral pathways for adults with CP resulted in limited joint working, poor communication between child and adult health services, and limited opportunities for young people to meet the adult team before leaving children’s services.

***Discharge letter to GP***

Quantitative and qualitative findings indicated that young people had limited contact with their GP. Few young people and parents knew that their GP had received a discharge letter and, based on reports from health professionals, in many cases GPs were not sent a discharge letter. Young people and parents described a reliance on their Paediatrician for medical queries, which negated the need to establish a relationship with their GP during childhood. This negatively affected their healthcare management in adulthood when they relied on their GP to coordinate care with limited knowledge of their history. Health professionals acknowledged that GP’s played an important role in coordinating care and encouraged families to build relationships with their GP in childhood.

***Life skills training***

Quantitative and qualitative findings indicated a lack of opportunity for formal life-skills training in thinking about and planning for the future. Young people wanted training and support across various life areas, including living options, employment options, access to assistive technology, and personal assistants, but described not receiving the support to meet their needs. They highlighted the potential value of peer support and role models in developing life skills, but many had limited opportunities to connect with others with CP.

### Dissonance between quantitative and qualitative findings

***Appropriate parent involvement***

Quantitative findings indicated parents were involved at a level that was appropriate to parents and young people. However, they also indicated that young people and parents were often not asked about how much each wanted the parent involved in care. Qualitative findings provided a nuanced interpretation of the causes and consequences of parent involvement, suggesting that the level of parent involvement was deemed appropriate by young people and parents in a health system that was not meeting the needs of either. Although young people appreciated their parent’s support in managing their healthcare, some wanted autonomy to express themselves in appointments and be treated as capable partners. However, they also wanted parents present when attending children and adult services to translate information, advocate for them, and be respected and listened to by health professionals. While parents recognised the importance of empowering their children to manage their healthcare, they were reluctant to relinquish control, wanting to achieve the best outcome during their limited time with health professionals. A high level of parent involvement did not prepare young people or parents for their changing role in adult services. Although some were forewarned about this change, the shift in responsibility felt abrupt and imposed on them by the new structure of adult health services.

### Silence between quantitative and qualitative findings

There was silence between quantitative and qualitative findings for the following practices.

***Promotion of health self-efficacy***

Promotion of health self-efficacy was addressed in quantitative but not qualitative findings. Most young people did not receive help to promote health self-efficacy even though the majority of health professionals said they provided this help. However, very few health professionals reported having a written policy for promoting health self-efficacy. Young people with more severe motor impairment were less likely to receive enough help to increase health self-efficacy compared to those with less severe motor impairment. Health professionals recognised the importance of supporting young people to self-manage and develop autonomy, and viewed self-efficacy as an implicit part of self-management. However, there was limited description of approaches to specifically target self-efficacy as a mechanism for improving a young person's ability to self-manage.

***Senior manager***

Promotion of health self-efficacy was addressed in quantitative but not qualitative findings. Most health professionals said there was not senior manager with responsibility for championing, implementing, monitoring, and reviewing the effectiveness of transition strategies and policies in their organisation. Qualitative findings did not describe a senior manager. They indicated that responsibility for championing and implementing transition strategies fell to individuals who were passionate about the importance of transition or were particularly skilled and knowledgeable in this area, resulting in unsustainable and inequitable care.

## Implications

Quantitative and qualitative findings from the Ignition Study primarily offered complementary information that frequently indicated transition practices were not adequately met. Qualitative findings served to elucidate the reasons why young people’s experience of transition was poor and provide some explanation for differences between young people’s and health professionals’ perceptions of transition. The integrated findings lead to some overarching implications, which were further informed by details of individual studies and discussions with our advisory groups of health professionals, young people, and parents ( Box 2). Implications are discussed in the context of current research.

**Box 2. Implications of the research**

1.  Tailoring parent involvement requires intentional actions to enable parents and young people to adapt to changing roles.

2.  A standardised transition pathway is required for all young people with CP across organisations, developed in collaboration with young people and parents.

3.  Provision of information to all young people, families and health professionals should be provided in a collaborative and phased approach that starts well before transfer.

4.  A common understanding of self-management is needed between young people, health professionals and parents.

5.  In the absence of health services for adults with CP that take a lifespan approach, there is a need for joint working between child services, adult services, and GP’s to optimise transition.

     1. Tailoring parent involvement requires intentional actions to enable parents and young people to adapt to changing roles.

Involving parents in their child’s healthcare at a level that is appropriate to young people and parents should be a priority during adolescence because it is associated with improved outcomes in adulthood ^19^. This requires organisations to adopt developmentally appropriate healthcare (DAH), which meets the biopsychosocial needs of young people, and a change in how health professionals engage both young people and parents ^20^. Our findings indicate that that parents need education and support from professionals and peers throughout their child's life to understand strengths-based ideas about health and development, and to learn how to adapt communication and collaboration with health professionals according to their child’s development. Parents and health professionals also have to concurrently provide young people with the time and space to build confidence to self-advocate, learn about their condition and healthcare, and practice communicating with health professionals. The mind and skill set of staff who are not ready to introduce DAH in both child and adult-oriented healthcare settings acts as a barrier ^20^. Organisations must create opportunities and develop capability among health professionals to tailor how they involve parents by supporting changes in organisational processes such as longer appointment times and introducing protocols and training. An organisational-wide change to support DAH requires formal support at senior executive and board level, planning that engages senior managers in both child and adult services, and an organisation wide strategy for training ^21^.

     2. A standardised transition pathway is required for all young people with CP across organisations, developed in collaboration with young people and parents.

A transition pathway should consist of a phased and systematic approach to transition, begin at a consistent age across organisations, include assessment of transition readiness and signposting, and be tailored to meet the needs of individuals, while ensuring that all young people and parents are automatically included in the pathway regardless of their perceived need. Each young person should be assigned a named worker with knowledge of the transition pathway to oversee the process. This person or people require access to standardised information and training to ensure consistency in young people’s and parents’ experience. Lack of standardised national practice regarding the age of transfer and an absence of written transition protocols is not unique to CP in Ireland ^22^. Variation in practice, resulting in inequity of care to young people, has also been reported for young people with diabetes, cystic fibrosis and congenital heart disease in Ireland ^23^.

Organisations need to create a dedicated role or provide several health professionals with dedicated time within their existing role to ensure that all young people have a named worker for transition and that this role is sustainable. In addition, one person at a senior level within organisations should have responsibility for implementing the transition pathway, advocating for evidence-based transition practices, and identifying and developing organisational processes, supports and training required to deliver organisation-wide DAH for young people. However, transition is often not a priority in health systems, which translates to resource limitations and lack of organisation-wide implementation of best-practice ^24^. Without formal support from management, a few enthusiastic and experienced individuals will continue to provide transitional care in isolation, perpetuating inequities in skills and experience across staff and inequity of care to young people ^21^.

     3. Provision of information to all young people, families and health professionals should be provided in a collaborative and phased approach that starts well before transfer.

A standardised approach to providing information that addresses a range of topics is needed to ensure that all young people receive information, not just those who ask for it or have a particular type or severity of impairment. Although health professionals may be concerned about the practicality of finding time to complete and discuss transition plans ^25^, a lack of standardised approach results in young people and parents having to advocate for support and source information, which may or may not be accurate. Parents of young people with cystic fibrosis in Ireland reported similar unmet information needs ^26^. In the UK, only 6% of young people with CP received a written transition plan compared to 26% with diabetes, suggesting unmet information needs is higher for people with CP than those with long-term medical conditions ^25^. Young people and parents identified the need for information about transition, CP, services and supports available in adulthood, education options, living options, mental health supports, managing finances, personal assistants, and rights and entitlements. These were similar to the information needs of people with CP identified in other countries ^27, 28^. Health professionals identified the need for information about a transition pathway and a directory of services that are available to adults with CP. To facilitate information sharing, one organisation needs to take responsibility for producing a reliable and updateable resource to share information and signpost individuals to trusted information.

     4. A common understanding of self-management is needed between young people, health professionals and parents.

Self-management encompasses diverse knowledge and skills that young people want and need to manage their physical and mental health and navigate health services as adults. To effectively support young people to develop self-management behaviours, they must be given knowledge and opportunities to practise skills within routine care in a phased but direct approach to ensure they recognise it as self-management support. When young people are given space to explore their boundaries to develop independence, they must be told that this approach is being taken and when and where to seek support if needed. This may be supplemented by structured programmes to develop skills and knowledge that young people want to manage their physical and mental health in adulthood. Such programmes have shown some evidence in improving self-management, condition knowledge and transition readiness ^29, 30^. To effectively self-manage, some young people may need additional support with self-advocacy, self-esteem and management of emotions. Further, promoting health self-efficacy should be prioritised as a distinct intervention that supports self-management but is not interchangeable with it, given the association between the promotion of health self-efficacy and improved outcomes in adulthood ^19^.

     5. In the absence of health services for adults with CP that take a lifespan approach, there is a need for joint working between child services, adult services, and GP’s to optimise transition. 

Meeting the adult team before transfer is associated with improved outcomes in adulthood ^19^. A gradual handover to adult services would facilitate nuanced information transfer about the young person, reassure families of support in adulthood, and allow the young person to connect to their new adult team. At a minimum, young people and parents should receive a joint meeting with professionals from child and adult health services. This requires child and adult health service professionals taking responsibility for transition. In the current context, providing a period of joint working is not possible for most young people with CP because health services available to adults with CP provide a limited service to address acute needs and have different, often narrower, eligibility criteria than children’s services. A study from the UK similarly identified that adult services generally have higher thresholds for accessing services ^24^. This lack of equivalent services for people with CP in adulthood may explain why fewer young people with CP have the opportunity to meet an adult team than young people with long-term medical conditions ^25^. Creating health services for adults with CP that take a lifespan approach is essential to enable joint working and optimise transition. In the absence of this, a systematic approach to involving GP’s in transition is required. Further research on the best approach to involving GP’s is required as there is currently limited evidence underpinning primary care interventions to improve transition outcomes ^31^. Although the GP is the most frequently visited health professional among adults with CP ^4^, young people and parents described challenges with obtaining satisfactory care from GP’s following discharge from children’s services. To adequately support adults with CP, GP’s need support to develop a basic knowledge of CP and the ability to work in partnership with adults. Adults with CP must also have sufficient knowledge and skills to work in partnership with GP’s and other professionals in adult health services to optimise the services currently available to them.

### Limitations

We cannot directly compare experiences of young people, parents and health professionals because health professional who participated may not have worked in the services that the young people and parents attended. People who participated in interviews may not have completed a questionnaire and vice versa, hence experiences from quantitative and qualitative data may not be from the same people. People with negative experiences and overestimation of difficulties with transfer to adult services may have been over-represented in both qualitative and quantitative data.

## Conclusion

Findings from the Ignition study provide insights into how transition may be improved for young people with CP. While findings highlight challenges that are unique to young people with CP or other child-onset physical disabilities, other challenges are applicable to people with different long-term conditions. Although provision of discrete supports such as standardised information, peer support and educational programmes about transition may partly improve the transition experience, systemic changes are required to facilitate the implementation of transitional care that adequately meets the needs of young people with CP. Transition will only be optimal for people with CP if multidisciplinary teams are created to provide proactive rather than reactive healthcare to adults and enable continuity of care from childhood to adulthood. This requires a fundamental shift in the perception of CP from a childhood condition to a child-onset, lifelong condition.

## Ethics and consent

This study obtained approval from the research ethics committee of the Principal Investigator’s host institution and the research ethics committees of two organisations that supported recruitment (Royal College of Surgeons in Ireland's Research Ethics Committee, REC201911010, 6 ^th^ February 2020; Enable Ireland Research, Ethics and Quality Committee, RA72JR2020, 16 ^th^ September 2020; Central Remedial Clinic Research Ethics Committee, 73001, 9 ^th^ July 2020. Written informed consent was not obtained from those who completed the online questionnaire because it was anonymous and completion indicated consent. However, before completing the anonymous online questionnaire, participants confirmed they had read and understood the information leaflet, knew they could withdraw at any time, and were happy to complete the questionnaire. Where a person completed a paper questionnaire, they provided written informed consent. Where the person was under the age of 18 years, their parent or guardian additionally provided written consent for their child to participate. Written or verbal informed consent was obtained from young people, parents, and health professional to participate in interviews, as approved by the ethics committees.

## Data Availability

Zenodo: Ryan J.Transition to adult services experienced by young people with cerebral palsy: A cross-sectional study (1.0) [Data set]. 2022. http://doi.org/10.5281/zenodo.6636481 ^32^. This project contains the following underlying data: “Transition_data”; quantitative data collected from surveys Data are available under the terms of the Creative Commons Attribution 4.0 International license (CC-BY 4.0) ( https://creativecommons.org/licenses/by/4.0/). Additional data that cannot be shared Data from interviews with participants cannot be sufficiently de-identified and participants did not give written consent for future use of their data. Therefore, supporting data is not available on request. Zenodo: Ryan J.Transition to adult services experienced by young people with cerebral palsy: A cross-sectional study (1.0) [Data set]. 2022 http://doi.org/10.5281/zenodo.6636481 ^32^. This project contains the following extended data: “metadata_v1”; questions and response options included in the questionnaires Data are available under the terms of the Creative Commons Attribution 4.0 International license (CC-BY 4.0) ( https://creativecommons.org/licenses/by/4.0/). Zenodo: Ryan, J (2024). Transition from child to adult health services for young people with cerebral palsy in Ireland; implications from a mixed-methods study: Extended data. https://doi.org/10.5281/zenodo.12567984 ^33^ This project contains the following extended data: GRAMMS; Good Reporting of A Mixed Methods Study checklist Table S1 Description of key transition practices and indicative interview questions Table S2 Convergence coding matrix displaying quantitative findings and qualitative findings Data are available under the terms of the Creative Commons Attribution 4.0 International license (CC-BY 4.0) ( https://creativecommons.org/licenses/by/4.0/).
