# Transition from child to adult health services for young people with cerebral palsy in Ireland; implications from a mixed-methods study

**DOI:** 10.12688/hrbopenres.13912.2

**Published:** 2025-01-31

**Authors:** Jennifer M. Ryan, Meriel Norris, Aisling Walsh, Amanda Breen, Owen Hensey, Claire Kerr, Sebastian Koppe, Grace Lavelle, Mary Owens, Michael Walsh, Thilo Kroll, Jennifer Fortune

**Affiliations:** 1 CP-Life Research Centre, School of Physiotherapy, RCSI University of Medicine and Health Sciences, Dublin, Ireland; 2 College of Health, Medicine and Life Sciences, Brunel University London, London, England, UK; 3 School of Population Health, RCSI University of Medicine and Health Sciences, Dublin, Ireland; 4 Central Remedial Clinic, Dublin, Ireland; 5 School of Nursing and Midwifery, Queen's University Belfast, Belfast, Northern Ireland, UK; 6 Enable Ireland, Galway, Ireland; 7 Institute of Psychiatry, Psychology & Neuroscience, King's College London, London, England, UK; 8 UCD IRIS Centre, School of Nursing, Midwifery and Health Systems, University College Dublin, Dublin, Leinster, Ireland

**Keywords:** cerebral palsy, disability, transition, health services, mixed-methods

## Abstract

**Background:**

Poor transition from child- to adult-oriented healthcare may lead to negative outcomes and dissatisfaction with services in adulthood. The aim of the study was to examine how transition is provided to and experienced by young people with cerebral palsy in Ireland. This report provides integrated quantitative and qualitative findings and implications based on the totality of knowledge generated.

**Methods:**

A convergent parallel mixed-methods study was conducted. Data were collected from people with cerebral palsy aged 16–22 years, parents, and health professionals using surveys and semi-structured interviews, which were both informed by a framework of nine key transition practices. Quantitative finding from the surveys and qualitative findings from interviews were integrated at the interpretation stage of the research using integration through joint displays. Implications were developed through discussions with health professionals, young people, and parents.

**Results:**

Surveys were completed by 75 young people/parents and 108 health professionals. Interviews were conducted with 13 young people, 14 parents, and 27 health professionals. There was complementarity between quantitative and qualitative findings indicating lack of a named worker, limited information provision, insufficient self-management support, no opportunity to meet the adult team, limited contact with the general practitioner, and no opportunity for attending formal life skills training. There was dissonance between quantitative and qualitative findings regarding appropriate level of parental involvement. Quantitative findings identified limited promotion of health self-efficacy and a lack of senior managers responsible for transition. These practices were not described in the qualitative findings.

**Conclusion:**

Implications of integrated findings include the need for a standardised transition pathway, intentional actions to enable parents and young people to adapt to changing roles, provision of information in a collaborative and phased approach, a common understanding of self-management between young people, parents and health professionals, and the need to involve general practitioners in transition.

## Introduction

Cerebral palsy (CP) is one of the most prevalent disabling conditions among children worldwide ^1^. Children with CP require transfer from child-centred to adult-oriented healthcare. In Ireland, this typically occurs between the age of 16 and 18 years. Transfer is the event when responsibility for healthcare shifts from a child to adult healthcare provider. After transfer to adult-oriented healthcare, many adults with CP experience difficulty navigating services ^2^, reduced access and visits to specialist services, and a decrease in use of rehabilitation services ^2, 3^. Poor preparation for this transfer may contribute to the deterioration in function and development of chronic conditions ^4, 5^.

While the term ‘transfer’ refers to the actual event of moving from child-centred to adult oriented healthcare, transition is defined as ‘the purposeful, planned process that addresses the medical, psychosocial, educational and vocational needs of adolescents and young adults with chronic medical and physical conditions as they move from child-centred to adult-oriented healthcare systems’ ^6^. Although guidance to support transition has been published in several countries ^7– 9^, there is still evidence that young people with CP and parents are not prepared for transition, lack support and information, and struggle to navigate the adult healthcare system ^3^.

To facilitate the implementation of successful transition for young people with CP, it is important to understand the experience of transition from multiple perspectives of young people with CP, parents and health professionals. In this paper, we report the integrated findings from the Ignition study, a mixed-methods study which aimed to examine how transition care is provided to and experienced by young people with cerebral palsy (CP) in Ireland ^10^. The Ignition study firstly assessed gaps in the current management of transition against best practice from the perspective of service users (young people and parents) and service providers through a quantitative survey. Quantitative findings indicated that many young people did not experience practices that may improve transition experiences and outcomes ^11^. Secondly, the Ignition study explored transition experiences from the perspective of young people with CP, their families and health professionals using semi-structured interviews. Qualitative findings suggested that the experience of transition is impacted by a number of diverse factors at several ecological levels ^12^. In-depth findings from quantitative and qualitative studies are separately reported elsewhere ^11– 13^. The purpose of this paper is to enhance the value of the study by integrating quantitative and qualitative findings and to provide implications for practice based on the totality of knowledge generated.

## Methods

We conducted a convergent parallel mixed methods study ^10^ and collected quantitative and qualitative data from young people with CP aged 16–22, parents, and health professionals. We used convenience and snowball sampling. We shared information about the quantitative and qualitative components of the study through three national organisations that provide health and social care services to people with CP, disability officers in higher education, special education needs schools, professional bodies, and social media. Participants could choose to take part in either the quantitative or qualitative components of the study, or both. As recruitment occurred through multiple streams, anonymised data was collected via online surveys, and consent was sought separately for each data collection method, it is not possible to identify whether there was overlap between those who took part in the quantitative and qualitative components. Data collection and analysis are described in detail elsewhere ^10– 13^. Data collection and analysis were informed by a framework of nine key transition practices representing best practices, which is published in the study protocol ^10^ and presented in Box 1. These transition practices were based on a research programme conducted in the UK ^14^. the UK NICE guideline on transition ^7^ and national strategies relating to children and neurodisability in Ireland ^15– 18^.

**Box 1. Description of nine key transition practices**

**Table:** 

**Named worker:** A named worker, known to the young person, oversees, co-ordinates or delivers transition support and acts as the link between the young person and the various practitioners involved in their care, including their GP, who is typically the main provider of primary care in the community. This person may not be formally allocated and may not be a health provider but should remain in close contact with health services.
**Appropriate parent involvement:** Involvement of parent(s)/carer(s) in the young person’s care at a level that is deemed appropriate by both the young person and parent(s)/carer(s)
**Information:** Child and adult services provide young people and families with information that describes the transition process and the support available before and after transfer. The information should specifically mention health services, which encompasses services that directly impact people's physical health, mental health, and social well-being. This information should be provided early enough to allow young people time to reflect and discuss with parent(s)/carer(s) or health professionals and be in an accessible format. Where there is no adult service for a young person to transfer to, information about known and trusted voluntary organisations who could provide support should be provided to the young person.
**Promotion of health self-efficacy:** Promotion of health self-efficacy (ie, actively helping young people to feel confident in managing their condition), where health encompasses complete physical, mental and social wellbeing, including provision of information to the young person about their condition and encouragement to take responsibility for their health.
**Self-management support:** Promotion of opportunities for self-management, where the individual is directly involved in planning and decision-making around their needs and takes responsibility for maintaining optimal physical, mental and social wellbeing.
**Meet the adult team:** A health professional from the relevant adult services or primary care meets the young person before they transfer from child services
**Senior manager:** A senior manager with responsibility for implementing transition strategies and policies, including facilitating good working relationships between child and adult services, ensuring appropriate materials are available, and monitoring that the person has a suitable appointment in adult services. This person may not be known to the young person
**Discharge letter to GP:** Where there is no adult service for a young person to transfer to, a detailed discharge letter is sent to the young person’s GP.
**Formal life-skills training:** Formal training, relevant to health condition, in wider life skills—education, gaining employment, finances, housing, social relationships, sexual health, mental health. The health service may not provide such training, but during consultations, staff should inquire about such matters and make referrals to other agencies as needed.

Quantitative data were collected using two questionnaires completed by 75 young people and/or parents and 108 health professionals ^11^. Young people had the option of completing the questionnaire alone, or with support from a parent. In instances where it was not possible to obtain the young person’s perspectives, parents could complete the questionnaire on their behalf. This was explained in the introduction to the questionnaire where it stated young people were encouraged to complete it with a parent, family member or carer, but could complete it alone or a parent or carer could complete it behalf of the young person if they were unable to. The questionnaire were developed by the research team in collaboration with and piloted by young people, parents, and health professionals. Minor changes were made to the in response to feedback from the piloting phase. We collected data on socio-demographic and condition-specific data, and the experience or provision of transition practices. Questions regarding the transition process were based on the framework of nine key transition practices representing best practices ^10^. Questions in the young person/ parent questionnaire mirrored those in the health professional questionnaire. For example, young people were asked if they had a named worker to help with the transition process, while health professionals were asked whether their service ensured that a named worker was provided. Descriptive statistics (e.g. mean, SD) were used to describe data as appropriate.

Qualitative data were collected using in-depth semi-structured interviews with 13 young people, 14 parents, and 27 health professionals ^12^. Young people and parents had the option to participate in interviews together or separately. For young people over 18 years, interviews were organised based on the young person’s preference to participate alone or with a parent. All young people under 18 years were interviewed together with a parent or guardian. The framework of key transition practices was used to develop the interview topic guides, and interview questions were thus similar to those asked in the questionnaires. Separate topic guides were developed for service users and service providers. These were piloted by young people, parents, and health professionals. Indicative questions are included in Table S1. Data were analysed using the Framework Method.

This study obtained approval from the research ethics committee of the Principal Investigator’s host institution and the research ethics committees of two organisations that supported recruitment (Royal College of Surgeons in Ireland's Research Ethics Committee, REC201911010, 6 ^th^ February 2020; Enable Ireland Research, Ethics and Quality Committee, RA72JR2020, 16 ^th^ September 2020; Central Remedial Clinic Research Ethics Committee, 73001, 9 ^th^ July 2020. Written informed consent was not obtained from those who completed the online questionnaire because it was anonymous and completion indicated consent. However, before completing the anonymous online questionnaire, participants confirmed they had read and understood the information leaflet, knew they could withdraw at any time, and were happy to complete the questionnaire. Where a person completed a paper questionnaire, they provided written informed consent. Where the person was under the age of 18 years, their parent or guardian additionally provided written consent for their child to participate. Written or verbal informed consent was obtained from young people, parents, and health professional to participate in interviews, as approved by the ethics committees.

### Integration

We integrated quantitative and qualitative data at the interpretation stage of the research using integration through joint displays ^19^. Key transition practices were used as a framework for theme development. Firstly, interview and questionnaire data was searched for data related to each theme. At this point, additional emerging themes were also identified. We then produced a convergence coding matrix by grouping themes according to similar concepts and interpreting grouped themes to produce meta-themes. The convergence matrix displayed quantitative and qualitative findings relating to each key transition practice in the framework (Table S2). An abbreviated version of the convergence matrix is presented in Table 1. Qualitative findings are presented according to themes from two individual papers ^12, 13^. Both qualitative papers were developed from interviews with young people with CP, parents, and health professionals. One paper explored factors influencing the young person’s experience of transition to adult health services at multiple ecological levels, while the other explored the experiences of young people during their transition to adulthood more broadly across several areas of life. Co-authors (JR, JF, MN, AW) discussed the matrix and considered where there was agreement and disagreement between findings, defined as convergence (i.e., findings agree directly), complementarity (i.e., findings offer complimentary information), dissonance (i.e., findings seemingly contradict each other) or silence (i.e., themes arise in quantitative data or qualitative data but not both). Additionally, we identified the key implications of our integrated findings through discussions with the wider research team and our advisory groups of health professionals, young people, and parents. Therefore, in this paper, we report the integrated findings and present implications of this research.

**Table 1.** Abbreviated convergence matrix.

Key Transition practice	Quantitative findings ^11^	Summary of key qualitative findings	Convergence/complementarity/dissonance/silence
Named worker	36% of young people reported having a named worker who supports the transition process 35% of health professionals reported that named workers were provided or available at their service.	Young people expressed frustration at the lack of guidance and direction from knowledgeable professionals ^13^ Service providers acknowledged a structured transition process as best practice but high staff turnover, long waiting lists and competing priorities meant transition was a low priority ^12^ Transition support roles were typically informal, and each professional typically took responsibility for their own piece ^12^ Without dedicated roles, there was a lack of clarity about who is responsible for the transition process ^12^ Coordination often relied on a dedicated, passionate individual strategizing and driving the process, which was unsustainable if this person moved on 12	Complementarity
Appropriate parent involvement	90% of young people reported appropriate parent involvement 81% of parents reported that they were involved in the young person’s care at an appropriate level 66% of health professionals consulted young people about parent involvement. 63% of health professionals consulted parents about parent involvement.	Health professionals frequently communicated with parents in children’s services, leaving young people feeling disempowered ^13^ Health professionals in adult services primarily communicated with young people, who often sought parental presence to translate information, advocate, and provide emotional support ^13^ Young people felt more respected by health professionals when accompanied by their parents ^13^ During transition, some parents struggled to adjust to a shift in their roles from active decision-makers to supportive/ consultative ^13^ Prior to transfer, most health professionals did not seek the opinion of young people or parents regarding parental involvement ^13^ A phased approach, where young people attended parts of appointments alone, increased their confidence and helped them adapt to the adult system ^13^	Dissonance
Information provision	24% young people received information around transition 69% of health professionals reported providing information around transition Young people post-discharge were more likely to receive information than young people pre-discharge (40% vs 6%)	Health professionals saw their role as facilitative rather than prescriptive ^13^ Limited access to reliable information about transition limited young people's ability to engage with the transition process ^12^ The transfer felt sudden and abrupt for some young people. Discharge appointments sometimes came without notice ^12^ Young people and parents desired access to transparent information and reassurance of service continuation ^12^ Young people expressed frustration as they were left seeking information, rather than it being offered to them ^12^ Health professionals felt limited in their ability to provide information, advocate for adult services and provide reassurance due to their limited awareness of existing services, uncertainty in the process and difficulty sourcing information ^12^	Complementarity
Promotion of health self-efficacy	37% of young people reported the promotion of health self-efficacy 73% of health professionals reported the promotion of health self-efficacy Young people in GMFCS level III and V were less likely to receive enough help to improve their health self-efficacy compared to level I	Health professionals acknowledged that the protected environment of children’s services could lead to dependence ^13^ Health professionals emphasised the significance of respecting young people's choices ^13^ Post-transfer, young people aimed to gain control and experience managing their CP. However, limited understanding of their CP posed challenges participating in healthcare ^13^ Parents highlighted the importance of tailored treatment sessions that empowered their children and granted them a sense of control over their healthcare journey ^13^	Silence
Self-management support	36% of young people reported self-management support for physical health: 17% of young people reported self-management support for mental health 73% of health professionals reported that self-management support was provided or available at their service	Young people recommended that health professionals encourage their participation by directing questions towards them, offering choices, involving them in decision-making, and supporting communication ^13^ During the transition to adulthood, young people took on more responsibility, such as managing exercise routines and daily tasks ^13^ Health professionals were surprised at how little young people knew about their condition and ageing with CP ^13^ Some young people faced challenges due to low confidence in communicating and advocating for themselves with new healthcare teams ^13^ Parents appreciated the support of health professionals in fostering self-management and independence skills in their children ^13^ To encourage self-management, health professionals personalised treatment, involved young people in decision-making, promoted positive body awareness, and encouraged collaborative goal setting ^13^ Young people and health professionals agreed that increased psychological support would enhance the transition to adulthood and promote self-acceptance ^13^	Complementarity
Meet the adult team	16% of young people reported meeting the adult team 30% of health professionals reported that young people had the opportunity to meet the adult team Young people post-discharge more likely to have opportunity to meet adult team than people pre-discharge (25% vs 6%)	Health professionals and parents highlighted an ideal transition would include a defined handover period with joint working between paediatric and adult providers ^12^ Health professionals described limited formal contact and opportunities for connection between adult and children’s services ^12^ Service providers described friction between child and adult services ^12^ Health professionals felt that without follow-up by adult services, preparation completed by children's service providers was fruitless ^12^ Health professionals in adult services felt that children’s services could do more to send appropriate referral letters, prepare young people for adulthood, and improve awareness of what adult services could offer ^12^ Young people and parents felt that opportunities to become familiar with adult services provided reassurance, reduced anxiety, and facilitated new working relationships ^12^	Complementarity
Senior manager	20% of health professionals reported that there was a senior manager responsible for transition at their service	Health professionals from children’s services felt that attempts at initiating transitional care interventions floundered due to lack of time and dedicated staffing ^12^ Health professionals believed a dedicated transition coordinator role would reduce uncertainty and clarify responsibility ^12^ Health professionals felt that a dedicated role, with dedicated time attached to it, would enable a person to take ownership of and proactively plan and drive the process ^12^ In addition to a coordinator, health professionals highlighted the need for a person to provide oversight of the process, act as a bridge between services, and advocate for evidence-based transition practice within and across organisations ^12^	Silence
Discharge letter to GP	10% of young people were aware that their GP had received a discharge letter 56% of health professionals sent a discharge letter to the young person’s GP	Health professionals and parents valued paediatrician oversight to offer comprehensive care coordination ^12^ The absence of a paediatrician equivalent in adult services meant young people and families must coordinate their care and navigate pathways to access services in an unfamiliar and fragmented system ^12^ Young people at all GMFCS levels, who were both pre- and post-discharge, reported limited interaction with their GP in childhood ^12^ Paediatricians attempted to grow the connection between service users and GPs through good communication channels throughout the young person’s childhood ^12^ Health professionals and parents highlighted that general healthcare and therapy in adulthood should be community centred with referral to specialists or hospitals when required ^12^	Complementarity
Life skills training	16% of young people reported receiving formal skills training 36% of health professionals reported that formal skills training was provided or available at their service	Young people desired more training and support across life areas such as living and employment options, access to assistive technology, and personal assistants ^13^ Young people frequently highlighted the need for more support in areas such as confidence, interpersonal skills, self-acceptance, self-esteem, and management of anxiety and emotional well-being ^13^ Parents and health professionals acknowledged the value of immersive environments, such as overnight trips and respite, for young people to develop life skills in supportive settings ^13^ Parents and health professionals noted that skills like advocacy and resilience were often given less priority than conventional life skills training ^13^ Most young people and parents, particularly those in GMFCS level I, desired opportunities to connect with others with CP to receive support, reassurance, and share experiences and strategies ^13^	Complementarity

## Results

We outline integrated findings for each transition practice according to whether there was complementarity, dissonance, or silence between quantitative and qualitative findings.

### Complementarity between quantitative and qualitative findings

There was complementarity between quantitative and qualitative findings for the following practices.

***Named worker***

Quantitative and qualitative findings indicated lack of a named worker to oversee, co-ordinate or deliver transition support. Most young people did not have a named worker, and most health professionals stated their service did not ensure each young person had a named worker for transition. Limited time and high staff turnover restricted health professionals’ ability to support young people with transition within their existing roles. Within some services, an individual with an interest and passion for transition took on the role of a named worker, resulting in variation within and between services. As illustrated by the following quote, this became unsustainable when the person did not have dedicated time to fulfil the role:

“A good few years back, there was a little bit of a push. One of the paediatricians, since retired, had an interest and we started. We kind of started a type of a transition. Sort of. We got together anyway and we tried, but it kind of fell by the wayside after she retired.” (physiotherapist)

In the absence of a dedicated named worker for transition assigned to young people, staff also lacked clarity about who was responsible for supporting transition.

***Information provision***

Quantitative and qualitative findings indicated young people and families received limited information describing the transition process and the support available before and after transfer. Young people who had been discharged from children’s services were more likely to receive information than those still attending children’s services. Receiving information immediately before discharge, or in many cases not at all, prevented young people from engaging in transition and resulted in young people and parents feeling abandoned. One young person described their experience with their discharge meeting as follows:

“I didn't even realize that a meeting was something that you did. I was being asked did I have any questions at this team meeting. I think it was just a bit of a shock to see them [paediatric health professionals]. Because I hadn't seen them in a bit. I didn't have time to think of any questions that I might have now.” (young person)

Health professionals often provided information when asked and when they perceived it was needed. They saw their role as facilitative rather than prescriptive. Further, health professionals sometimes withheld information to protect the young person and family from a lack of equivalent health services in adulthood. They also hesitated to discuss the young person’s condition or medical history with them if they thought parents had not yet shared this information. In contrast, young people often preferred to receive information directly and found it difficult to find trusted sources of information. Young people and parents also had specific information needs and knowledge gaps that covered a breadth of topics, including the transition process, the causes and possible complications of their CP, service provision in adulthood, managing their CP in adulthood, and education and living options, which may be beyond one professional’s expertise.

***Self-management support***

Quantitative and qualitative findings indicated that young people did not receive enough support to self-manage their physical and mental health. Although most health professionals stated they or their service supported young people to take responsibility for maintaining optimal health, very few had a written protocol for this. Qualitative findings suggest that young people and health professionals have different perspectives on self-management. Young people received support to manage specific aspects of their care, such as completing an exercise programme. However, they wanted support to develop a range of skills and knowledge to enable them to manage their condition as adults, such as talking to health professionals, making appointments, and identifying when and how to access health services. One young person described how attending appointments independently in the years prior to transition prepared was beneficial for self-management in adulthood:

“I did my own thing from about 14, 15...if I had any issues or if my mam or dad had noticed anything, that would be at the start of the appointment and then they’d go get a coffee and I’d do my appointment I think it did prepare me for getting older” (young person)

However, young people often lacked time, space and opportunities in healthcare settings to practise and develop skills. Their lack of knowledge about their condition, medical history and potential changes in their condition as they age also negatively impacted their ability to self-manage. Health professionals provided space for young people to develop self-management skills. However they often did not explicitly make the young person aware of this, which resulted in the young person feeling unsupported.

***Meet the adult team***

Quantitative and qualitative findings indicated young people did not have the opportunity to meet with someone who provided their healthcare in adulthood before transfer. Qualitative findings indicated a lack of equivalent multidisciplinary services for adults with CP, which resulted in health professionals in children’s service not knowing if the young person would receive a service in adulthood or where to refer them. If they identified a service for the young person, they often did not know which professional within the service would see the young person. Many adults with CP accessed community-based or primary care services in adulthood, which typically accepted people with an acute need only. Therefore, it was impossible to directly transfer the young person from children’s services. These services also typically lacked expertise about the needs of adults with CP and were sometimes unwilling to engage with adults with CP. One health professional summarised this challenge as follows:

“There's nobody to refer clients onto at the moment. Some primary care teams will take them on and some won't, and generally speaking, the primary care is they need to have a need at that time to be seen, so I'll make the referral, but they're not going to necessarily meet them unless they need something at that time” (occupational therapist)

The lack of adult services and uncertainty about referral pathways for adults with CP resulted in limited joint working, poor communication between child and adult health services, and limited opportunities for young people to meet the adult team before leaving children’s services.

***Discharge letter to GP***

Quantitative and qualitative findings indicated that young people had limited contact with their GP. Few young people and parents knew that their GP had received a discharge letter and, based on reports from health professionals, in many cases GPs were not sent a discharge letter. Young people and parents described a reliance on their Paediatrician for medical queries, which negated the need to establish a relationship with their GP during childhood. This negatively affected their healthcare management in adulthood when they relied on their GP to coordinate care with limited knowledge of their history. One parent explained:

“suddenly your GP is in control of everything. Which is fine if you've got a really good GP, like we have. But the GPs don't always know the kids. Especially if they've been dealt with in services the whole time and they've had their paediatrician in the clinic looking after everything for them. And then you're suddenly going back to a GP who goes ‘but I don't actually know your child. I don't really know how I'm supposed to help you here.’ It's been a learning curve for us” (parent)

Health professionals acknowledged that GP’s played an important role in coordinating care and encouraged families to build relationships with their GP in childhood.

***Life skills training***

Quantitative and qualitative findings indicated a lack of opportunity for formal life-skills training in thinking about and planning for the future. Young people wanted training and support across various life areas, including living options, employment options, access to assistive technology, and personal assistants, but described not receiving the support to meet their needs. One young person described receiving a lack of practical guidance as follows:

“...it would have been great if she [health professional] could give me a few suggestions. If she would say “do you know, I think this would be good for you” because they know me for years, they know what type of person I am... But it was always...“oh just follow your gut and dream big”. It was really inspirational and all, but it’s not practical” (young person)

Young people also highlighted the potential value of peer support and role models in developing life skills, but many had limited opportunities to connect with others with CP.

### Dissonance between quantitative and qualitative findings

***Appropriate parent involvement***

Quantitative findings indicated parents were involved at a level that was appropriate to parents and young people. However, they also indicated that young people and parents were often not asked about how much each wanted the parent involved in care. Qualitative findings provided a nuanced interpretation of the causes and consequences of parent involvement, suggesting that the level of parent involvement was deemed appropriate by young people and parents in a health system that was not meeting the needs of either. Although young people appreciated their parent’s support in managing their healthcare, some wanted autonomy to express themselves in appointments and be treated as capable partners. However, they also wanted parents present when attending children and adult services to translate information, advocate for them, and be respected and listened to by health professionals. As illustrated by the following quote, at times the presence or absence of parents also impacted the duration of appointments:

“if your Mam’s not there to fight your corner, they just bully you...if Mam wasn’t there I’d get a 20 minute appointment. If Mam’s there I get 45 minutes. That’s why I make her come because I get more out of the session” (young person)

While parents recognised the importance of empowering their children to manage their healthcare, they were reluctant to relinquish control, wanting to achieve the best outcome during their limited time with health professionals. A high level of parent involvement did not prepare young people or parents for their changing role in adult services. Although some were forewarned about this change, the shift in responsibility felt abrupt and imposed on them by the new structure of adult health services.

### Silence between quantitative and qualitative findings

There was silence between quantitative and qualitative findings for the following practices.

***Promotion of health self-efficacy***

Promotion of health self-efficacy was addressed in quantitative but not qualitative findings. Most young people did not receive help to promote health self-efficacy even though the majority of health professionals said they provided this help. However, very few health professionals reported having a written policy for promoting health self-efficacy. Young people with more severe motor impairment were less likely to receive enough help to increase health self-efficacy compared to those with less severe motor impairment. Health professionals recognised the importance of supporting young people to self-manage and develop autonomy, and viewed self-efficacy as an implicit part of self-management. However, there was limited description of approaches to specifically target self-efficacy as a mechanism for improving a young person's ability to self-manage.

***Senior manager***

Promotion of health self-efficacy was addressed in quantitative but not qualitative findings. Most health professionals said there was no senior manager with responsibility for championing, implementing, monitoring, and reviewing the effectiveness of transition strategies and policies in their organisation. Qualitative findings did not describe a senior manager. They indicated that responsibility for championing and implementing transition strategies fell to individuals who were passionate about the importance of transition or were particularly skilled and knowledgeable in this area, resulting in unsustainable and inequitable care.

## Implications

Quantitative and qualitative findings from the Ignition Study primarily offered complementary information that frequently indicated transition practices were not adequately met. Qualitative findings served to elucidate the reasons why young people’s experience of transition was poor and provide some explanation for differences between young people’s and health professionals’ perceptions of transition. The integrated findings lead to some overarching implications, which were further informed by details of individual studies and discussions with our advisory groups of health professionals, young people, and parents. Implications are discussed in the context of current research. Based on the implications identified, a number of recommendations have been developed. Recommendations are presented alongside relevant implications in Table 2.

**Table 2.** Recommendations.

Implication	Recommendations
1. Tailoring parent involvement requires intentional actions to enable parents and young people to adapt to changing roles.	• Organisations should create opportunities for health professionals to tailor how they involve parents by supporting changes in organisational processes (e.g., longer appointment times; introducing protocols and training). • Organisations should adopt DAH through: (i) support at senior executive and board level; (ii) planning between senior managers in both child and adult services; (iii) developing and implementing an organisation-wide strategy for training.
2. A standardised transition pathway is required for all young people with CP across organisations, developed in collaboration with young people and parents.	• Organisations should ensure that all young people have a named worker for transition by: (i) creating dedicated, sustainable named worker roles; (ii) providing health professionals with dedicated time to act as a named worker within their existing roles. • Organisations should identify one person at a senior level to take responsibility for the transition process at an organisational level, including: (i) implementing a transition pathway; (ii) advocating for evidence-based transition practices; (iii) identifying and developing relevant organisational processes, supports and training.
3. Provision of information to all young people, families and health professionals should be provided in a collaborative and phased approach that starts well before transfer.	• Organisations should develop a standardised approach to providing information relevant to transition to all young people and families, not only those who ask for it or have a particular type or severity of impairment. • Health professionals should have access to a transition pathway and a directory of services that are available to adults with CP. • To facilitate information sharing, one organisation needs to take responsibility for producing a reliable and updateable resource to share information and signpost individuals to trusted information.
4. A common understanding of self-management is needed between young people, health professionals and parents.	• Organisations should implement structured programmes for young people to develop the skills and knowledge needed to manage one’s own physical and mental health in adulthood. • Young people should be given opportunities to learn about and practice self-management. It is crucial that when young people are given space to develop their independence/ self-management skills, they are: (i) informed that this approach is being taken; (ii) when and where to seek support if needed.
5. In the absence of health services for adults with CP that take a lifespan approach, there is a need for joint working between child services, adult services, and GP’s to optimise transition.	• Handover from child to adult services should be gradual to: (i) facilitate nuanced information transfer about the young person; (ii) reassure families of support in adulthood; (iii) allow the young person to connect to their new adult team. • At a minimum, young people and parents should receive a joint meeting with professionals from child and adult health services. • GPs should be encouraged to develop a basic knowledge of CP and the ability to work in partnership with adults with CP. • Adults with CP must also have sufficient knowledge and skills to work in partnership with GPs and professionals in adult health services to optimise the services available to them.

     1. Tailoring parent involvement requires intentional actions to enable parents and young people to adapt to changing roles.

Involving parents in their child’s healthcare at a level that is appropriate to young people and parents should be a priority during adolescence because it is associated with improved outcomes in adulthood ^20^. This requires organisations to adopt developmentally appropriate healthcare (DAH), which meets the biopsychosocial needs of young people, and a change in how health professionals engage both young people and parents ^21^. Our findings indicate that parents need education and support from professionals and peers throughout their child's life to understand strengths-based ideas about health and development, and to learn how to adapt communication and collaboration with health professionals according to their child’s development. Parents and health professionals also have to concurrently provide young people with the time and space to build confidence to self-advocate, learn about their condition and healthcare, and practice communicating with health professionals. The mind and skill set of staff who are not ready to introduce DAH in both child and adult-oriented healthcare settings acts as a barrier ^21^. Organisations must create opportunities and develop capability among health professionals to tailor how they involve parents by supporting changes in organisational processes such as longer appointment times and introducing protocols and training. An organisational-wide change to support DAH requires formal support at senior executive and board level, planning that engages senior managers in both child and adult services, and an organisation wide strategy for training ^22^.

     2. A standardised transition pathway is required for all young people with CP across organisations, developed in collaboration with young people and parents.

A transition pathway should consist of a phased and systematic approach to transition, begin at a consistent age across organisations, include assessment of transition readiness and signposting, and be tailored to meet the needs of individuals, while ensuring that all young people and parents are automatically included in the pathway regardless of their perceived need. Each young person should be assigned a named worker with knowledge of the transition pathway to oversee the process. This person or people require access to standardised information and training to ensure consistency in young people’s and parents’ experience. Lack of standardised national practice regarding the age of transfer and an absence of written transition protocols is not unique to CP in Ireland ^23^. Variation in practice, resulting in inequity of care to young people, has also been reported for young people with diabetes, cystic fibrosis and congenital heart disease in Ireland ^24^.

Organisations need to create a dedicated role or provide several health professionals with dedicated time within their existing role to ensure that all young people have a named worker for transition and that this role is sustainable. In addition, one person at a senior level within organisations should have responsibility for implementing the transition pathway, advocating for evidence-based transition practices, and identifying and developing organisational processes, supports and training required to deliver organisation-wide DAH for young people. However, transition is often not a priority in health systems, which translates to resource limitations and lack of organisation-wide implementation of best-practice ^25^. This may be a significant barrier to the development and implementation of a standardised transition pathway. Without formal support from management, a few enthusiastic and experienced individuals will continue to provide transitional care in isolation, perpetuating inequities in skills and experience across staff and inequity of care to young people ^24^.

     3. Provision of information to all young people, families and health professionals should be provided in a collaborative and phased approach that starts well before transfer.

A standardised approach to providing information that addresses a range of topics is needed to ensure that all young people receive information, not just those who ask for it or have a particular type or severity of impairment. Although health professionals may be concerned about the practicality of finding time to complete and discuss transition plans ^26^, a lack of standardised approach results in young people and parents having to advocate for support and source information, which may or may not be accurate. Parents of young people with cystic fibrosis in Ireland reported similar unmet information needs ^27^. In the UK, only 6% of young people with CP received a written transition plan compared to 26% with diabetes, suggesting unmet information needs is higher for people with CP than those with long-term medical conditions ^26^. Young people and parents identified the need for information about transition, CP, services and supports available in adulthood, education options, living options, mental health supports, managing finances, personal assistants, and rights and entitlements. These were similar to the information needs of people with CP identified in other countries ^28, 29^. Health professionals identified the need for information about a transition pathway and a directory of services that are available to adults with CP. To facilitate information sharing, one organisation needs to take responsibility for producing a reliable and updateable resource to share information and signpost individuals to trusted information.

     4. A common understanding of self-management is needed between young people, health professionals and parents.

Self-management encompasses diverse knowledge and skills that young people want and need to manage their physical and mental health and navigate health services as adults. To effectively support young people to develop self-management behaviours, they must be given knowledge and opportunities to practise skills within routine care in a phased but direct approach to ensure they recognise it as self-management support. When young people are given space to explore their boundaries to develop independence, they must be told that this approach is being taken and when and where to seek support if needed. This may be supplemented by structured programmes to develop skills and knowledge that young people want to manage their physical and mental health in adulthood. Such programmes have shown some evidence in improving self-management, condition knowledge and transition readiness ^30, 31^. To effectively self-manage, some young people may need additional support with self-advocacy, self-esteem and management of emotions. Further, promoting health self-efficacy should be prioritised as a distinct intervention that supports self-management but is not interchangeable with it, given the association between the promotion of health self-efficacy and improved outcomes in adulthood ^20^.

     5. In the absence of health services for adults with CP that take a lifespan approach, there is a need for joint working between child services, adult services, and GP’s to optimise transition. 

Meeting the adult team before transfer is associated with improved outcomes in adulthood ^20^. A gradual handover to adult services would facilitate nuanced information transfer about the young person, reassure families of support in adulthood, and allow the young person to connect to their new adult team. At a minimum, young people and parents should receive a joint meeting with professionals from child and adult health services. This requires child and adult health service professionals taking responsibility for transition. In the current context, providing a period of joint working is not possible for most young people with CP because health services available to adults with CP provide a limited service to address acute needs and have different, often narrower, eligibility criteria than children’s services. A study from the UK similarly identified that adult services generally have higher thresholds for accessing services ^25^. Although CP is a lifelong condition, it has often historically been considered to be a paediatric condition, which may also impact the development of policy and provision of services for adults. For example, CP is included in the National Model of Care for Paediatric Healthcare Services in Ireland ^18^ but not in the National Policy and Strategy for the Provision of Neuro-Rehabilitation Services ^16^. In Ireland, disability services for children are typically provided by multidisciplinary teams, consisting of professionals with various areas of expertise who thus have the capability to provide support for a broad range of needs. In contrast, there are less available multidisciplinary services for adults with disability, limiting the scope of the support provided ^32^. This lack of equivalent services for people with CP in adulthood may explain why fewer young people with CP have the opportunity to meet an adult team than young people with long-term medical conditions ^26^. Expanding the provision of multidisciplinary supports for adults would require significant dedicated funding. However, this has been identified as a key objective in increasing service capacity in the Irish disability sector ^32^.

Creating health services for adults with CP that take a lifespan approach is essential to enable joint working and optimise transition. In the absence of this, a systematic approach to involving GP’s in transition is required. Further research on the best approach to involving GP’s is required as there is currently limited evidence underpinning primary care interventions to improve transition outcomes ^33^. Although the GP is the most frequently visited health professional among adults with CP ^4^, young people and parents described challenges with obtaining satisfactory care from GP’s following discharge from children’s services. To adequately support adults with CP, GP’s need support to develop a basic knowledge of CP and the ability to work in partnership with adults. Adults with CP must also have sufficient knowledge and skills to work in partnership with GP’s and other professionals in adult health services to optimise the services currently available to them.

### Limitations

We cannot directly compare experiences of young people, parents and health professionals because health professional who participated may not have worked in the services that the young people and parents attended. People who participated in interviews may not have completed a questionnaire and vice versa, hence experiences from quantitative and qualitative data may not be from the same people. People with negative experiences and overestimation of difficulties with transfer to adult services may have been over-represented in both qualitative and quantitative data.

## Conclusion

Findings from the Ignition study provide insights into how transition may be improved for young people with CP. While findings highlight challenges that are unique to young people with CP or other child-onset physical disabilities, other challenges are applicable to people with different long-term conditions. Although provision of discrete supports such as standardised information, peer support and educational programmes about transition may partly improve the transition experience, systemic changes are required to facilitate the implementation of transitional care that adequately meets the needs of young people with CP. Transition will only be optimal for people with CP if multidisciplinary teams are created to provide proactive rather than reactive healthcare to adults and enable continuity of care from childhood to adulthood. This requires a fundamental shift in the perception of CP from a childhood condition to a child-onset, lifelong condition.

## Ethics and consent

This study obtained approval from the research ethics committee of the Principal Investigator’s host institution and the research ethics committees of two organisations that supported recruitment (Royal College of Surgeons in Ireland's Research Ethics Committee, REC201911010, 6 ^th^ February 2020; Enable Ireland Research, Ethics and Quality Committee, RA72JR2020, 16 ^th^ September 2020; Central Remedial Clinic Research Ethics Committee, 73001, 9 ^th^ July 2020. Written informed consent was not obtained from those who completed the online questionnaire because it was anonymous and completion indicated consent. However, before completing the anonymous online questionnaire, participants confirmed they had read and understood the information leaflet, knew they could withdraw at any time, and were happy to complete the questionnaire. Where a person completed a paper questionnaire, they provided written informed consent. Where the person was under the age of 18 years, their parent or guardian additionally provided written consent for their child to participate. Written or verbal informed consent was obtained from young people, parents, and health professional to participate in interviews, as approved by the ethics committees.

## Data Availability

Zenodo: Ryan J. Transition to adult services experienced by young people with cerebral palsy: A cross-sectional study (1.0) [Data set]. 2022. http://doi.org/10.5281/zenodo.6636481 ^32^. This project contains the following underlying data: “Transition_data”; quantitative data collected from surveys Data are available under the terms of the Creative Commons Attribution 4.0 International license (CC-BY 4.0) ( https://creativecommons.org/licenses/by/4.0/). Additional data that cannot be shared Data from interviews with participants cannot be sufficiently de-identified and participants did not give written consent for future use of their data. Therefore, supporting data is not available on request. Zenodo: Ryan J. Transition to adult services experienced by young people with cerebral palsy: A cross-sectional study (1.0) [Data set]. 2022 http://doi.org/10.5281/zenodo.6636481 ^34^. This project contains the following extended data: “metadata_v1”; questions and response options included in the questionnaires Data are available under the terms of the Creative Commons Attribution 4.0 International license (CC-BY 4.0) ( https://creativecommons.org/licenses/by/4.0/). Zenodo: Ryan, J (2024). Transition from child to adult health services for young people with cerebral palsy in Ireland; implications from a mixed-methods study: Extended data. https://doi.org/10.5281/zenodo.12567984 ^35^ This project contains the following extended data: GRAMMS; Good Reporting of A Mixed Methods Study checklist Table S1 Description of key transition practices and indicative interview questions Table S2 Convergence coding matrix displaying quantitative findings and qualitative findings Data are available under the terms of the Creative Commons Attribution 4.0 International license (CC-BY 4.0) ( https://creativecommons.org/licenses/by/4.0/).
