# Supplementary material for: Transition from child to adult health services for young people with cerebral palsy in Ireland; implications from a mixed-methods study
Source: HRB Open Res. 2025 Jan 31;7:47. Originally published 2024 Jul 17. [Version 2] doi: 10.12688/hrbopenres.13912.2 (PMC11875073; doi:10.12688/hrbopenres.13912.2)
Supplement: Peer review history

# Reviewer report: Reviewer response for version 2

**DOI:** 10.21956/hrbopenres.15397.r46118

**Published:** 2025-03-04

**Authors:** Sandra Julsen Hollung

**Affiliations:** 1 Norwegian University of Science and Technology Faculty of Medicine and Health Sciences (Ringgold ID: 87362), Trondheim, Trøndelag, Norway; 2 Vestfold Hospital Trust, Tønsberg, Norway

This is a research study focusing on the transition experiences of young people with cerebral palsy (CP) in Ireland as they move from child to adult health services.

 The study employed a mixed-methods approach, integrating both quantitative and qualitative data from two previous published studies. Participants included young people, parents, and health professionals, creating a holistic view of the transition process. The research in this study was guided by a framework of nine key transition practices to further outline the provision of transition support, identifying areas of complementarity, dissonance, and silence between quantitative and qualitative findings. The findings call for actionable recommendations to enhance transition support, which could significantly improve the experiences of young people with CP as they move into adulthood.

 The manuscript has previously been reviewed by two reviewers who have highlighted several areas for improvement. Therefore, the authors have already made several valuable improvements to the manuscript accordingly. The presentation of the manuscript has been strengthened, and the findings have been effectively communicated.

 I simply have some minor comments.

 First, as new text has been added to the latest version of the manuscript, there are a few incomplete sentences and grammatical issues to be corrected as follows:

 1. Abstract, Methods:

 “Quantitative finding” should be plural?

 2. Methods, first paragraph, last two sentences:

 I believe this should be one sentence, please note punctuation after UK:

  “These transition practices were based on a research programme conducted in the UK ^14^. the UK NICE guideline on transition ^7^ and national strategies relating to children and neurodisability in Ireland ^15–18^.”

 3. Methods, second paragraph:

 Please note missing the word “on” between “it behalf”:

 “This was explained in the introduction to the questionnaire where it stated young people were encouraged to complete it with a parent, family member or carer, but could complete it alone or a parent or carer could complete it behalf of the young person if they were unable to.”

 4. Methods, second paragraph:

 Should “questionnaire” be plural?

 5. Methods, second paragraph:

 Minor changes were made to the … ?

 “Minor changes were made to the in response to feedback from the piloting phase.”

 6. Methods, third paragraph:

 “Young people and parents had the option to participate in interviews together or separately. For young people over 18 years, interviews were organised based on the young person’s preference to participate alone or with a parent. All young people under 18 years were interviewed together with a parent or guardian.”

 This is a response to a previous reviewer.

 However, it is still a bit unclear as the first sentence states that all young people and parents had the option to participate together or separately … then later, all under 18 years were interviewed together with a parent or guardian.

 Question: Did youths under 18 years also have the option to participate separately, but all chose to participate with a parent/guardian? Or was this option not available to them?

 7. Results, Self-management, first paragraph, last sentence:

 I believe the word “prepared” should be removed?

 “One young person described how attending appointments independently in the years prior to transition prepared was beneficial for self-management in adulthood.”

 8. Results, Senior manager:

 The first sentence appears to be incorrectly copied from the previous paragraph? Suggest to delete.

 Other general comments:

1. Throughout the paper (methods, results and acknowledgments), there is mention of advisory groups (health professionals, youths and parents/Young Person Advisory Group and the Parent Advisory Group). Because they participated in the interpretation of the findings, it would be helpful to have more detail about these groups. Perhaps they were connected to the two previous studies, and therefore a reference could be added?
2. This novel and significant paper elucidates the need for and suggests potential improvements in the transition from child to adult health care services. The findings and recommendations are highly relevant not only to youths with CP in Ireland, but also to those in other countries, highlighting the *universal* importance of enhancing transitional care." Perhaps consider a sentence on generalizability in the discussion section?

Is the work clearly and accurately presented and does it cite the current literature?

Yes

If applicable, is the statistical analysis and its interpretation appropriate?

Yes

Are all the source data underlying the results available to ensure full reproducibility?

Yes

Is the study design appropriate and is the work technically sound?

Yes

Are the conclusions drawn adequately supported by the results?

Yes

Are sufficient details of methods and analysis provided to allow replication by others?

Yes

Reviewer Expertise:

cerebral palsy, epidemiology, lifespan surveillance, health services research

I confirm that I have read this submission and believe that I have an appropriate level of expertise to confirm that it is of an acceptable scientific standard.

# Reviewer report: Reviewer response for version 2

**DOI:** 10.21956/hrbopenres.15397.r45228

**Published:** 2025-02-27

**Authors:** Cristina Sarmiento

**Affiliations:** 1 Department of Physical Medicine and Rehabilitation, University of Colorado Anschutz, Aurora, CO, USA

Thank you for your thoughtful revisions and responses. This is important work that makes a meaningful contribution to the challenging issue of transition to adult health services for young people with cerebral palsy. I particularly appreciated the abbreviated joint display and inclusion of quotes from qualitative data as strengthening this manuscript.

Is the work clearly and accurately presented and does it cite the current literature?

Yes

If applicable, is the statistical analysis and its interpretation appropriate?

Yes

Are all the source data underlying the results available to ensure full reproducibility?

Partly

Is the study design appropriate and is the work technically sound?

Yes

Are the conclusions drawn adequately supported by the results?

Yes

Are sufficient details of methods and analysis provided to allow replication by others?

Partly

Reviewer Expertise:

Cerebral palsy, lifespan care, healthcare transitions, health services research

I confirm that I have read this submission and believe that I have an appropriate level of expertise to confirm that it is of an acceptable scientific standard.

# Reviewer report: Reviewer response for version 2

**DOI:** 10.21956/hrbopenres.15397.r45227

**Published:** 2025-02-04

**Authors:** Gavin Colquitt

**Affiliations:** 1 Appalachian State University, Boone, North Carolina, USA

Thank you for your hard work in addressing my comments. This paper is much improved and highlights important findings that will hopefully be very impactful on the system in which the study was conducted.

Is the work clearly and accurately presented and does it cite the current literature?

Yes

If applicable, is the statistical analysis and its interpretation appropriate?

Partly

Are all the source data underlying the results available to ensure full reproducibility?

Yes

Is the study design appropriate and is the work technically sound?

Yes

Are the conclusions drawn adequately supported by the results?

Yes

Are sufficient details of methods and analysis provided to allow replication by others?

Partly

Reviewer Expertise:

cerebral palsy, community-based participatory research

I confirm that I have read this submission and believe that I have an appropriate level of expertise to confirm that it is of an acceptable scientific standard.

# Reviewer report: Reviewer response for version 1

**DOI:** 10.21956/hrbopenres.15260.r41395

**Published:** 2024-09-03

**Authors:** Cristina Sarmiento

**Affiliations:** 1 Department of Physical Medicine and Rehabilitation, University of Colorado Anschutz, Aurora, CO, USA

This is a mixed methods study integrating quantitative and qualitative data to examine how transition is provided to and experienced by young people with CP in Ireland. While the qualitative and quantitative findings from this study have previously been published, this study integrates the findings and proposes clinical and programmatic implications based on the integrated findings. Overall, this is a highly relevant article with strong methodology and clear clinical relevance. 

 I would suggest the following in order to clarify and strengthen this paper:

 Introduction: While I appreciate that the qualitative and quantitative findings are described elsewhere, I recommend briefly providing information on what each of these studies examined and found. This will help clarify what this mixed methods paper adds above and beyond the two previously published studies.

 Methods: While the quantitative and qualitative studies are published elsewhere, there are a few key details that should be provided in this mixed methods manuscript:

 - It is important to clarify whether or not there was overlap in the participants who completed the survey questionnaires and those who completed the interviews. Were these the same participants? Could a participant complete one portion of the study, but not the other? Who made that decision?

 - Additionally, how did the study team decide if a parent vs young adult with CP would complete the survey, or participate in the interview?

 - What questionnaires were used? Are these validated survey measures, or developed by the study team?

 - Under "Integration", the authors state, "Qualitative findings are presented according to themes from two individual papers." How do these two individual papers differ from each other? Would recommend briefly stating this.

 Results:

 - Was there no convergence between findings?

 - While the joint display is provided in supplemental material, given the importance of the joint display to the mixed methods results, I recommend adding an abbreviated version of the joint display to the manuscript itself. At present, the manuscript does not provide evidence of quantitative (ie, statistical results) or qualitative (ie, quotations) data as foundation for its integrated, summative results. Again, while these are separately published, some of these data should be included in the mixed methods manuscript to justify the presented results.

 - I responded "partly" to the question, "Are all the source data underlying the results available to ensure full reproducibility?" The qualitative data are not available due to confidentiality concerns. I believe this increases the need to provide quotations from the qualitative data in this manuscript.

 Discussion/Implications: 

 - For audiences unfamiliar with the role of a GP and how this differs from the rest of the care team or a pediatrician, consider adding a brief description of this role.

 - Several of the implications provided would require large policy changes and/or significant dedicated funding (eg, longer appointment times, organization-wide training, health professionals with dedicated time to coordinate transitions, joint visits, creation of new healthcare services). While these are all important, appropriate, and ideal suggestions, funding is a significant barrier to many of them. I would suggest discussing potential solutions to this, or an acknowledgement of this as a significant barrier.

 - The authors raise an interesting point in implication #5, where they describe that adult CP services have narrower eligibility criteria and provide more limited care than pediatric services. Why is this the case? Is this a target for policy change? Why or why not?

Is the work clearly and accurately presented and does it cite the current literature?

Yes

If applicable, is the statistical analysis and its interpretation appropriate?

Yes

Are all the source data underlying the results available to ensure full reproducibility?

Partly

Is the study design appropriate and is the work technically sound?

Yes

Are the conclusions drawn adequately supported by the results?

Yes

Are sufficient details of methods and analysis provided to allow replication by others?

Partly

Reviewer Expertise:

Cerebral palsy, lifespan care, healthcare transitions, health services research

I confirm that I have read this submission and believe that I have an appropriate level of expertise to confirm that it is of an acceptable scientific standard, however I have significant reservations, as outlined above.

# Response

**Published:** 2025-01-26

**Authors:** Jennifer M. Ryan

**Affiliations:** Royal College of Surgeons in Ireland, Ireland

Thank you for your time and constructive feedback on this paper. We have a​​​​​​​dressed each comment below under section headings.

**Introduction**

**Comment 1: **I recommend briefly providing information on what each of these studies examined and found.

**Response 1: **Thank you for this recommendation. We have added brief information on what each of these studies examined and found in the final paragraph of the introduction section.

**Methods**

**Comment 1: **It is important to clarify whether or not there was overlap in the participants who completed the survey questionnaires and those who completed the interviews. Were these the same participants? Could a participant complete one portion of the study, but not the other? Who made that decision?

**Response 1: **Participants in this study had the opportunity to participate in either element of the study, or both, at their own discretion. We have clarified this in the manuscript. However, it is not possible for the research team to identify whether there was overlap between participants in the qualitative and quantitative components, as recruitment occurred through multiple streams and anonymised data was collected via online surveys. We sought consent separately for each data collection method (i.e. survey and interview). We have added this information to the manuscript.

**Comment 2: **How did the study team decide if a parent vs young adult with CP would complete the survey, or participate in the interview?

**Response 2: **Young people in this study were given the option to decide whether they wanted to complete the questionnaire independently or with support from a parent. We have added that at the start of the questionnaire, it stated young people were encouraged to complete it with a parent, family member or carer, but could complete it alone. While the questionnaire was designed to be accessible for young people with all levels of impairment, we acknowledged that there may be instances where it was not possible to obtain the young person's perspective. In these instances, parents were invited to complete the questionnaire with their young person on their young person’s behalf. This was also stated in the introduction to the questionnaire. In the interview component of the study, young people and parents could participate in interviews together or separately. This was organised based on the young person’s preference for young people who were over 18. All young people under 18 took part in an interview together with a parent. We have adapted the manuscript to include this information.

**Comment 3: **What questionnaires were used? Are these validated survey measures, or developed by the study team?

**Response 3: **The questionnaires used were developed by the study team in collaboration with young people with CP, parents, and health professionals. We have added information to clarify this in the manuscript.

**Comment 4: **Under "Integration", the authors state, "Qualitative findings are presented according to themes from two individual papers." How do these two individual papers differ from each other? Would recommend briefly stating this

**Response 4: **Thank you for this recommendation. One of these papers specifically explored factors influencing the young person’s experience of transition to adult services at multiple ecological levels. The second explored the experiences of young people during their transition to adulthood more broadly. We have adapted the integration section to briefly state this.

**Results**

**Comment 1: **Was there no convergence between findings.

**Response 1: **No, through the integration process, we identified instances of complementarity, dissonance, and silence between the studies, but we did not identify instances of convergence.

**Comment 2: **I recommend adding an abbreviated version of the joint display to the manuscript itself.

**Response 2: **Thank you for this suggestion. We have added an abbreviated version of the convergence matrix into the manuscript. In this table, we summarise quantitative and qualitative findings related to each transition process and highlight whether there was convergence, complementarity, dissonance, or silence between findings.

**Comment 3: **The qualitative data are not available due to confidentiality concerns. I believe this increases the need to provide quotations from the qualitative data in this manuscript.

**Response 3: **Thank you for this feedback. We have added representative quotes to support the findings where relevant.

**Discussions/Implications**

**Comment 4: **For audiences unfamiliar with the role of a GP. Consider adding a brief description of this role.

**Response 4: **Thank you for this suggestion. We have clarified that GPs are typically the main providers of primary care in the community in Box 1 (description of nine key transition practices).

**Comment 5: **Several of the implications provided would require large policy changes and/or significant dedicated funding. I would suggest discussing potential solutions to this, or an acknowledgement of this as a significant barrier.

**Response 5: **Thank you for this suggestion. We have adapted the manuscript to acknowledge instances where policy changes/ the need for dedicated funding may act as a barrier.

**Comment 6: **The authors raise an interesting point in implication #5, where they describe that adult CP services have narrower eligibility. Why is this the case? Is this a target for policy change? Why or why not?

 ​​​​​​​ **Response 6: **As there is limited publicly available information regarding the decision making process behind this difference in service provision, we cannot say with certainty why this is the case. However, existing research and policy publications point to a historic view of cerebral palsy as a paediatric condition and a lesser focus on the provision of multidisciplinary support for adults than children in the Irish disability context. We believe that both of these factors likely contribute to the narrower eligibility in adult CP services. The provision of more multidisciplinary supports for adults has been identified as a key objective in increasing service capacity by the Irish government. We have added this information to the manuscript.

# Reviewer report: Reviewer response for version 1

**DOI:** 10.21956/hrbopenres.15260.r41399

**Published:** 2024-07-25

**Authors:** Gavin Colquitt

**Affiliations:** 1 Appalachian State University, Boone, North Carolina, USA

This paper presents implications from the Ignition study. It is well-designed and addresses a critical need in the area of transition services. The paper also has the strength of including multiple perspectives, such as those of young people with CP, parents/caregivers, and health professionals. The following recommendations for each section could greatly strengthen the paper and increase its impact.

**Abstract**

 Each term should be written out the first time it is mentioned before using an abbreviation.

 More detail on the survey methods would be helpful.

 Check for punctuation errors in this section.

 The phrase “there was silence” is unclear. Similarly, the phrase “with quantitative findings indicating these were not present for most young people, but qualitative findings not describing these practices” is also unclear. I understand that this comes from the convergence coding matrix, but additional context would be helpful in the abstract.

**Introduction**

 The first paragraph should be moved to the end of this section to clearly state the research question.

 A reference is needed to support the incidence rates of CP.

 The sentence “Children with CP require transfer from child-centered to adult-oriented healthcare, typically between the age of 16 and 18 years” needs context. Is this referencing typical practice in Ireland?

 In the third paragraph, it would be helpful to differentiate between ‘transfer’ and ‘transition.’

**Methods**

 Provide additional information on the reliability and validity of the survey questions used, particularly regarding their validation for parents, providers, and young people with CP.

 More information is needed on the specific surveys and interview guides. The type of analysis in the paper requires knowledge of specific aspects of these questions, including which questions were asked in the survey and the interviews. This is crucial for understanding the results through the lens of complementarity, dissonance, and silence.

 In the paragraph on integration, the process for identifying the findings needs additional detail. Please detail the steps on how this was a systematic process.

**Results**

 In the paragraph discussing the lack of a Named Worker, there are several references to ‘staff.’ What role is this referring to, and how does it differ from a health professional?

 In the paragraph on Information Provision, the reference to ‘their CP’ needs context. It seems to refer to specific symptoms and types of CP, but this needs clarification.

**Implications**

 This section effectively makes clear recommendations from the Ignition study. However, taking out the recommendations and creating a subsection dedicated to them would be helpful. This section contains valuable information, but the recommendations are somewhat lost. If the recommendations were pulled out and placed in a separate section, it would be more impactful. Perhaps even consider a separate box linking implications to specific recommendations.

Is the work clearly and accurately presented and does it cite the current literature?

Yes

If applicable, is the statistical analysis and its interpretation appropriate?

Partly

Are all the source data underlying the results available to ensure full reproducibility?

Yes

Is the study design appropriate and is the work technically sound?

Yes

Are the conclusions drawn adequately supported by the results?

Yes

Are sufficient details of methods and analysis provided to allow replication by others?

Partly

Reviewer Expertise:

cerebral palsy, community-based participatory research

I confirm that I have read this submission and believe that I have an appropriate level of expertise to confirm that it is of an acceptable scientific standard, however I have significant reservations, as outlined above.

# Response

**Published:** 2025-01-26

**Authors:** Jennifer M. Ryan

**Affiliations:** Royal College of Surgeons in Ireland, Ireland

Thank you for your time reviewing this paper and constructive feedback. We have outlined our response to each comment below under the section headings.

**Abstract**

**Comment 1: **Each term should be written out the first time it is mentioned before using an abbreviation.

**Response 1: **Thank you for highlighting this issue. We have reviewed the abstract and removed abbreviations from the abstract.

**Comment 2: **More detail on the survey methods would be helpful.

**Response 2: **We appreciate this feedback. However we are constrained by the word count allowance for this abstract, we have adapted the wording to clarify that like the interviews, the surveys were informed by a framework of nine key transition practices and that quantitative findings came from the surveys.

**Comment 3: **Check for punctuation errors in this section.

**Response 3:** Thank you for this suggestion. We have checked for punctuation errors and edited accordingly.

**Comment 4: **The phrase “there was silence” is unclear. Similarly, the phrase “with quantitative findings indicating these were not present for most young people, but qualitative findings not describing these practices” is also unclear.

**Response 4: **Thank you for this feedback. We have reworded this section to improve clarity. It now reads ‘Quantitative findings identified limited promotion of health self-efficacy and a lack of senior managers responsible for transition. These practices were not described in the qualitative findings’.

**Introduction**

**Comment 1: **first paragraph should be moved to the end of this section to clearly state the research question

**Response 1: **We appreciate this suggestion and agree that moving this paragraph enhances this section. We have moved this paragraph to the end of the section as suggested to ensure that the research question is clearly stated at the end.

**Comment 2: **A reference is needed to support the incidence rates of CP.

**Response 2: **Thank you for this feedback. We have added a reference.

****

**Comment 3: **The sentence “Children with CP require transfer from child-centered to adult-oriented healthcare, typically between the age of 16 and 18 years” needs context. Is this referencing typical practice in Ireland?

**Response 3: **Yes, this statement is based on our knowledge and experience of typical practice for transfer from child to adult services in Ireland. We have added information to clarify that this statement is referring to typical practice in Ireland in the manuscript.

**Comment 4: **In the third paragraph, it would be helpful to differentiate between ‘transfer’ and ‘transition.

**Response 4: **Thank you for this feedback, we acknowledge that the use of these terms without explicitly differentiating between may cause confusion among readers. To address this, we have clarified the distinction between the terms ‘transfer’ and ‘transition’ in the manuscript, as suggested.

**Methods**

**Comment 1: **Provide additional information on the reliability and validity of the survey questions used, particularly regarding their validation for parents, providers, and young people with CP.

**Response 1: **Thank you for this suggestion. We have provided additional information regarding how the questionnaire was developed in collaboration with, and piloted by, young people, parents, and health professionals, and how the questionnaire was refined based on feedback from the pilot phase.

**Comment 2: **More information is needed on the specific surveys and interview guides.

**Response 2: **Thank you for this feedback. While we signpost to the individual published studies in the introduction section, which provide in-depth information about the data collection processes utilised, we agree that this manuscript benefits from more information on the data collection tools used. We have thus provided additional information on the questionnaires and interview guides in the manuscript as recommended. The questionnaires are available to access under extended data.

**Comment 3: **In the paragraph on integration, the process for identifying the findings needs additional detail. Please detail the steps on how this was a systematic process.

**Response 3: **Thank you for this feedback. We have provided additional detail about the systematic process used for identifying findings at the integration stage.

**Results**

**Comment 1: **paragraph discussing the lack of a Named Worker, there are several references to ‘staff.’ What role is this referring to, and how does it differ from a health professional?

**Response 1: **The term ‘staff’ is used in this instance to refer to the broader workforce in healthcare settings, including health professionals and professionals in other roles. This aligns with the description of nine key transition processes provided (Box 1), which explains that a named worker in this context may not always be a health provider.

**Comment 2: **paragraph on Information Provision, the reference to ‘their CP’ needs context. It seems to refer to specific symptoms and types of CP, but this needs clarification.

**Response 2: **Thank you for this suggestion. We have changed this to **‘**the causes and possible complications and consequences of their CP’ to provide greater clarity.****

**Implications**

**Comment 1: **Taking out the recommendations and creating a subsection dedicated to them would be helpful. This section contains valuable information, but the recommendations are somewhat lost. If the recommendations were pulled out and placed in a separate section, it would be more impactful. Perhaps even consider a separate box linking implications to specific recommendations.

**Response 1: **Thank you for this feedback. To ensure that the recommendations are clear, we have added a table (table 2) linking implications to specific recommendations.
